# Double-Gap Magnetic Flux Concentrator Design for High-Sensitivity Magnetic Tunnel Junction Sensors

**DOI:** 10.3390/s19204475

**Published:** 2019-10-15

**Authors:** Jiafei Hu, Minhui Ji, Weicheng Qiu, Long Pan, Peisen Li, Junping Peng, Yueguo Hu, Huiyan Liu, Mengchun Pan

**Affiliations:** 1 College of Intelligence Science and Technology, National University of Defense Technology, Changsha 410073, China; garfield_nudt@163.com (J.H.); minhuiji109@163.com (M.J.); plong_2017@163.com (L.P.); lips13@163.com (P.L.); junpingpeng@126.com (J.P.); pmc_nudt@vip.163.com (M.P.); 2 China Huayi Broadcasting Corporation, Fuzhou 350003, China; seablhy@163.com

**Keywords:** magnetic sensor, magnetic tunnel junction, magnetic flux concentrator, sensitivity

## Abstract

To improve the sensitivity of the magnetic tunnel junction(MTJ)sensor, a novel architecture for a double-gap magnetic flux concentrator (MFC) was studied theoretically and experimentally in this paper. The three-dimensional finite element model of magnetic flux was established to optimize the magnetic field amplification factor, with different gaps. The simulation results indicate that the sensitivity of an MTJ sensor with a double-gap MFC can be significantly better than that of a sensor with a traditional single-gap MFC, due to the fact that the magnetic magnification sharply increases with the decrease in effective gap width. Besides this, the half-bridge MTJ sensors with the double-gap MFC were fabricated using photolithography, ion milling, evaporation, and electroplating processes. Experimental results show that the sensitivity of the MTJ sensor increased by ten times compared to the sensor without the double-gap MFC, which underlines the theoretical predictions. Furthermore, there is no significant increase in the sensor noise. The work in this paper contributes to the development of high-performance MTJ sensors.

## 1. Introduction

The magnetic tunnel junction (MTJ) sensor is based on the effect of tunneling magnetoresistance (TMR), which has had a major role in biomedicine, industry, and geophysics due to its high sensitivity and low power consumption [1,2,3]. In order to reduce the noise, a lot of research on the design of magnetic multilayer thin films, optimization of the fabrication, and post-processing treatment has been done [4,5,6]. The structure of the Wheatstone bridge is commonly used in MTJ sensors [7]. It is well known that a full Wheatstone bridge can get a linear output, but the technological process is relatively complex due to the need for precise design of different sensitive axes. To simplify the production process, the structure of the half bridge is a reliable alternative to suppress noises, which includes two sensitive arms and two reference arms [8,9].

Besides this, many recent studies have focused on the enhancement of the sensor sensitivity. The MTJ sensor hybridized with a superconducting flux-to-field transformer was designed to detect the femtotesla field. However, this type of sensor requires cryogenic refrigeration to operate, and the sensitivity is proportional to the superconducting flux area [10,11]. Magnetic flux concentrators (MFC) are another competitive method to improve the properties of MTJ sensors [12,13,14,15,16]. Firstly, it is easy to realize industrial production with low cost and low power consumption by using the mature integrated circuit technology, which benefits the miniaturization of the sensor system. Drljaca et al. analyzed the impact of the shape of single-gap MFCs on magnification by numerical simulations and proposed that the magnification would increase 50% using the tanga concentrators instead of the simple bar-shaped ones [17]. Valadeiro et al. obtained up to 400 times gain in sensitivity by introducing a double-layer MFC with two different magnetic materials and a vertical tapering, demanding a complex microfabrication process [18]. Yin et al. used a micro-MFC combined with an external MFC to increase the signal of the MTJ bridge by a factor of 400 times [19].

In order to obtain a high-performance MTJ sensor with high magnetic sensitivity and low noise, this paper proposes a novel architecture of double-gap MFC designed to achieve an improved half-bridge sensitivity. The double-gap MFCs reduce the gap width effectively by adding a magnetic stripe in the gap area. The theoretical calculation shows that average magnetic magnification is about three times larger than that of the half bridge sensors with only single-gap MFC. Finally, an experimental sensitivity gain of ∼10× has been obtained in the sensor with the MFC length of 1300 μm and magnetic stripe width of 65 μm, which is consistent with the theoretical simulation results. Furthermore, the hysteresis characteristics of the sensor have been obviously suppressed and the noise level of the sensor remains nearly unchanged after the introduction of the double-gap MFC.

## 2. MFC Setup and Simulation Modeling

**Figure 1:**
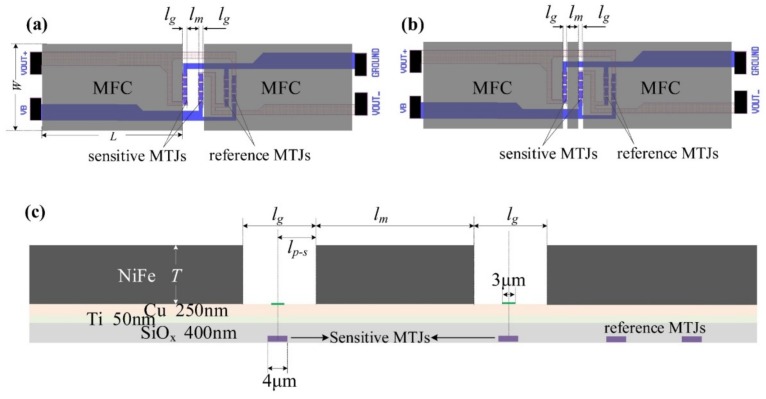
Layout of the sensor containing (**a**) single-gap magnetic flux concentrators (MFCs), (**b**) double-gap MFCs, and (**c**) cross-section layout highlighting the double gap area.

The schematic diagrams of the half Wheatstone bridge MTJ sensor with two types of MFC structures are displayed in Figure 1a (single-gap) and Figure 1b (double-gap). The MTJ sensor consisted of two parts: the bridge circuit, configured with four MTJ arms for sensing, and the soft magnetic MFC for magnifying the magnetic field [8]. In terms of the bridge circuit, the left two MTJ arms located in the MFC gap were sensitive MTJs and the other two reference MTJ arms were shielded by the concentrator (Figure 1a,b). Under this configuration, the resistance change of the reference arms responding to magnetic field could be negligible compared with the sensitive arms. Hence, the sensitivity of this sensor mainly depended on the magnetoresistance effect of the sensitive arms and the magnetic magnification of the MFC.

Compared with the single-gap MFC, the double-gap MFC of the sensor was constructed by inserting a magnetic strip between the two sensitive arms, as shown in Figure 1b. More details of the double-gap sensor are highlighted with the cross-section layout, as shown in Figure 1c. The two kinds of MFCs had the same length *L* and width *W*, with the only difference in the gap region.

For the double-gap MFC, the gap width as schematized in Figure 1c can be defined as:

(1) $$W_{double-gap}=l_{g},$$

in which *l*_g_ is determined by the size of the designed MTJ and the limitation of the electroplating process. For single-gap MFCs, the gap is mainly determined by the MTJ size and the spacing between two sensitive arms. In contrast to the double-gap MFC, the gap for single-gap MFC is expressed as:

(2) $$W_{single-gap}=2\times l_{g}+l_{m}$$

here, *l*_m_ is the width of the magnetic strip of double-gap MFC, which is determined by the distance between two sensitive arms and limited by the micro-fabrication method and technology.

In order to study the magnetic magnification of the two MFCs, the three-dimensional (3D) finite element modeling of MFC was developed using the AC/DC modules for electromagnetic analysis of the Comsol Multiphysics software. According to Maxwell’s equations, the mathematical model of the static magnetic field can be expressed as:

(3) $$-\nabla \cdot \left(\mu_{0}\left(\nabla A+M\right)\right)=0.$$

In the simulation, the MFC was designed with a length of *L* = 1300 μm, a width of *W* = 350 μm, and the thickness *T* varied from 1 to 10 μm. The width of the magnetic stripe (*l*_m_) in the double-gap MFC was designed as 65 μm, and the initial value of the gap width (*l*_g_) was set to 20 μm. Besides this, the distance between the pole and sensor center was *l*_p-s_ = 8 μm. To increase the simulation accuracy, the MFC was surrounded by a large area of air box, whose size was 8 × 8 × 4 mm^3^. Considering the sensor was mainly designed for detecting weak magnetic field, i.e., the MFC worked in the transition range, the magnetic permeability of the MFC could be regarded as a fixed value [17]. In this case, the relative permeability of the air and the soft magnetic area were set to 1 and 1000, respectively. It is worth pointing out that as the size of the MFC was much larger than that of the MTJs, the influence of the magnetic material of the MTJs could be neglected. To establish the external magnetic field environment, a static magnetic field *H*_0_ = (10,0,0) Oe was set at the air box, and the initial magnetic potential of the boundary was set to *A*_0_ = (0,0,0) Wb/m. The Free Triangular feature node, with a maximum element size of 1 × 10^−6^ m and 1 × 10^−5^ m, was used for mesh generation in the gap and MFC, respectively. Finally, a swept mesh and a Free Tetrahedra were applied for the entire structure.

According to the definition, the magnetic magnification of the MFC can be calculated by the ratio of the magnetic field in the MFC’s gap to the external magnetic field [14]. In our simulation, the magnetic field in the gap for both single and double gap MFC was computed by the average of the magnetic field in the gap’s middle area, with 3 μm width and 350 μm length, as marked by the green color in Figure 1c.

## 3. Discussion

**Figure 2:**
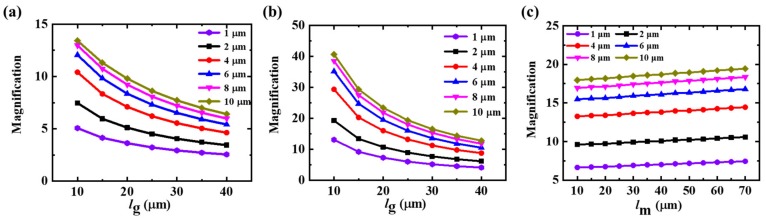
The magnification as a function of gap width with different concentrator thicknesses for (**a**) single-gap MFC, (**b**) double-gap MFC, and (**c**) the magnification as a function of magnetic stripe width under different concentrator thicknesses for double-gap MFC.

The effect of gap width on the magnetic magnification under different concentrator thicknesses was calculated, as shown in Figure 2. It is shown that the magnetic magnification decreased quickly with the increase in the gap width when the thickness of the MFC was constant. In addition, the increase in the thickness of the MFC could also enhance the magnetic magnification, which is consistent with the previous studies [20]. However, it is worth pointing out that the magnetic magnification would become stable when the thickness increases above a certain value. It is difficult to increase the magnetic magnification only by changing the thickness of magnetic concentrators. As a result, reducing the gap width is a more effective method. More importantly, the magnetic magnification of double-gap MFC can be evidently improved compared with that of the traditional singe-gap structure under the same *l*_g_. Taking *l*_g_ = 10 μm as an example, the double-gap MFC’s magnetic magnification could reach up to 19.4 times, while it was only 7.3 times for the single-gap MFC under *l*_g_ = 65 μm and *T* = 2 μm.

The influence of *l*_m_ on magnetic magnification was further studied. As shown in Figure 2c, the magnetic magnification of the double-gap MFC was not significantly improved with the increase in *l*_m_ (from 10 μm to 70 μm). Since *l*_m_ was much smaller than the length of the MFC (1300 μm), the magnetic magnification of the double-gap MFC was mainly determined by the shape and dimension of the MFC. Therefore, the width of the magnetic strip *l*_m_ had little influence on the magnification. Obviously, this result is beneficial to the design and fabrication of the double-gap MFC.

**Figure 3:**
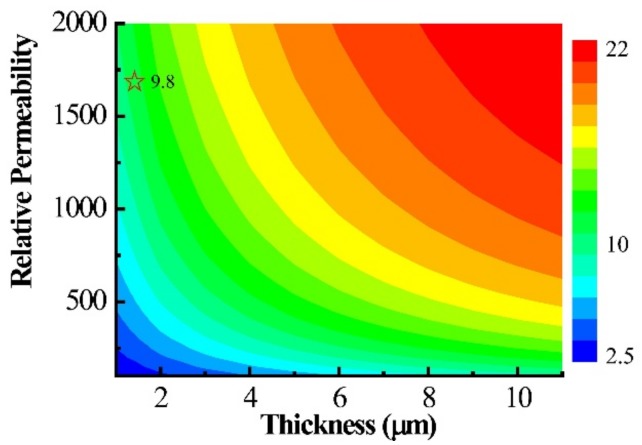
The magnetic magnification of the double-gap MFC (*l*_g_ = 20 μm) dependence on the relative permeability and MFC thickness.

In practice, the MFC is usually made of high-permeability materials, such as laminate NiFe and amorphous CoZrNb films [12], and the permeability of the soft magnetic film usually varies with the thickness of MFC. In order to study the effect of relative permeability (*µ*_r_) on the magnetic magnification, the magnification dependence on the relative permeability and MFC thickness was systematically simulated. The results are displayed in Figure 3. It is shown that the magnetic magnification increased with the MFC thickness, as well as the relative permeability. When the MFC thickness was 1.2 μm and the relative permeability was 1700, the magnetic magnification was 9.8 times, which is marked in Figure 3. This result provides a theoretical guidance for the subsequent experimental results.

## 4. Experimental Results

**Figure 4:**
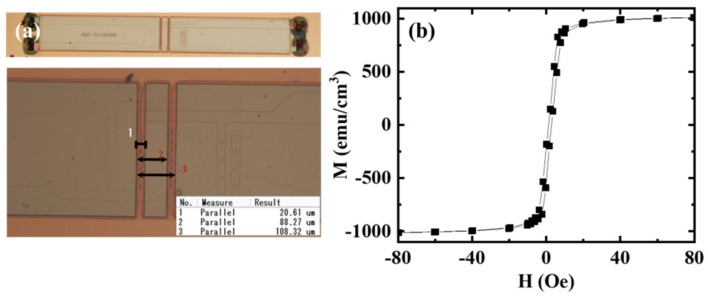
(**a**) Microscope image of the MTJ sensor with the double-gap MFC, (**b**) hysteresis loop of the soft NiFe film.

In order to verify this double-gap proposal, an MTJ half bridge sensor integrated with double-gap NiFe MFC was fabricated, which consisted of four MTJ bridge arms. Each bridge arm was designed with seven MTJ cells in series. Firstly, the MTJ multilayer film (Ta(5)/Ru(5)/Pt_40_Mn_60_(20)/Co_70_Fe_30_(3)/Ru(1)/Co_40_Fe_40_B_20_(2)/MgO(2)/Co_40_Fe_40_B_20_(2)/Ni_80_Fe_20_(10)/Ir_25_Mn_75_(10)/Ta(5)/capping layer (numbers in nanometer)) was stacked using UHV(Ultra-high vaccum) magnetron sputtering with a base vacuum of 1 × 10^−6^ Pa. A soft pinned free layer was designed to get a linear output response of the MTJ device. The structure of the half bridge with rectangle MTJs (4 μm × 8 μm) was then micron-pattern defined by the processes of photolithography, ion milling, and evaporation plating. After that, the whole half-bridge sensor was annealed at 340 °C under a magnetic field of 0.7 T for 1 h, with a vacuum of about 2 × 10^−4^ Pa. The TMR was expected to rise to 160% from 30%, and the pinned direction of the reference layer could be set to the short axis of the MTJ rectangle after the annealing. Before the electroplating, a SiO_x_ layer (400 nm) was deposited with ICP-CVD (inductively coupled plasma-chemical vapor deposition) for insulating the MTJ devices from the MFC, and then a Ti/Cu (50 nm/250 nm) metal layer (cathode electrode) was deposited with sputtering as the electroplating seed layer. After that, the electroplated regions (MFC) were patterned by standard photolithography with AZ4620 photoresist. Finally, the Ni_70_Fe_30_ films were electroplated using a customized wafer plating system at 1 A/dm^2^ and 50 °C. The anode was soluble compound material, consisting of pure Ni (99.7%) and Fe (99.95%), and the detailed chemical composition of the electrolyte was reported elsewhere [21]. The cross-section diagram of the double-gap MFC is shown in Figure 1c, and the typical microscopy image of the half bridge sensor with double-gap MFC is shown in Figure 4a. The relevant parameters of *l*_g_ (about 20.6 μm) and *l*_m_ (about 67.6 μm) were measured from the optical microscope. The concentrator thickness was 1.2 μm, measured by the step profiler DEKTAK 150. Figure 4b shows the magnetization curve of the electroplated NiFe measured by MPMS^®^3 (Quantum Design, San Diego, CA, USA). The magnetic characteristics of NiFe MFC with the coercivity (1.6 Oe) and saturation magnetization (1000 emu/cm^3^) were extracted from the magnetization curve. More importantly, a high relative permeability (1700) in a large range (about 9.8 Oe) was achieved.

**Figure 5:**
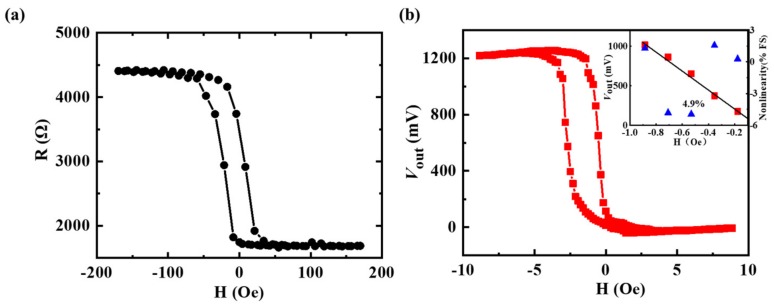
(**a**) R-H curve of one arm without the MFC, (**b**) output voltage of the magnetic sensor with the double-gap MFC in response to external magnetic field under the applied voltage of 2.5 V (the insert figure highlights the nonlinearity of the curve in the working range).

The general MFC designed for the half bridge could magnify the external magnetic field for the sensitive arms and shield the reference arms at the same time [22]. Therefore, the output voltage of the sensor without MFC could not respond to the external magnetic field, whose value was constantly equal to 0. The measurement of one arm of MTJs without MFC is an alternative to comparatively study the effect of MFC on the performance of the half bridge sensor. The sensitivity of the sensor without MFC could be calculated by the measurement of the R-H curve of one bridge arm. The resistance of one bridge arm versus the external magnetic field without double-gap MFC is illustrated in Figure 5a, and the output voltage of the magnetic sensor with the double-gap MFC in response to the external magnetic field under the applied voltage of 2.5 V is shown in Figure 5b.

The working range was defined on the right branch of the transfer curve and the linearity error (the maximum deviation (ΔY) from the measured curve and the fitted line as a percentage of the full-scale output (Y)) was less than 5%, as shown in the insert figure in Figure 5b. As reported previously, the sensitivity is defined as the percentage change in resistance (S = (1/*R*_0_)(dR)/(dH)), taking the sensitivity as the value of the slope of the right branch in the curve working range [23]. *R*_0_ represents the resistance of one bridge arm under zero magnetic field. The sensitivity of sensor without double-gap MFC extracted from the transfer curve slope was 4.6 %/Oe. For the case of the sensor with double-gap MFC, a higher sensitivity of 47.1 %/Oe was obtained. The sensitivity of the sensor with double-gap MFC was 10.2 times larger than that of the one without MFC. The experimental results coincide with the simulation results, which verifies the correctness of the simulation.

It is worth pointing out that hysteresis was still inevitable in the sensor, as shown in Figure 5, which is similar to previous papers [15,18]. In order to restrain the influence of this hysteresis for the application of sensing elements, on the one hand, researchers have taken many measures (e.g., optimizing the annealing process, biasing the sensor with permanent magnetic materials, or inducing perpendicular anisotropy in the free layer by reducing the thickness of the free layer [24,25]) to reduce the hysteresis, and on the other hand, we can also magnetize the sensor to the saturation state to ensure working at the same branch [26]. In this way, the hysteresis errors of the device without and with the double-gap MFC were 5.1% and 5.6%, respectively.

**Figure 6:**
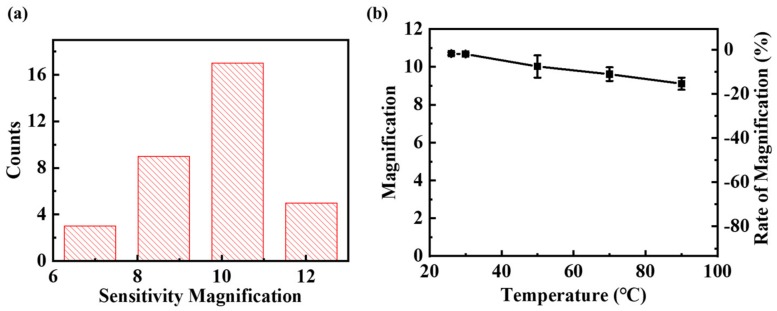
(**a**) The statistical result of the sensitivity magnification for sensors with and without the double-gap MFC, and (**b**) the sensitivity magnification versus temperature within the range of room temperature (26 °C) to 90 °C.

Figure 6a shows the sensitivity magnification of all measured sensors, and the results indicate that most of the sensors had a magnification of 9 to 11, and the magnifications exhibited an approximately normal distribution, which proves the reproducibility of the result. What’s more, sensitivity magnification changing with the temperatures is shown in Figure 6b, and the figure shows that the magnification decreased slowly with the temperature, and the magnification reduced to 9.1 when the temperature was 90 °C, which is about 18% lower than the result under room temperature (26 °C).

**Figure 7:**
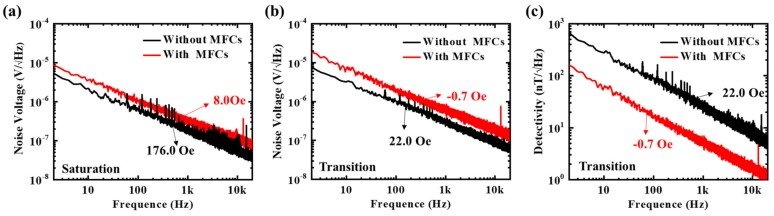
Noise spectra of the MTJ sensor with and without the double-gap MFC at (**a**) saturation state and (**b**) transition, (**c**) the detectivity of the MTJ sensor with the double-gap MFC at a DC magnetic bias field, which results in highest sensitivity, the magnetic field marked in the figure indicates the external magnet for noise measurement.

Furthermore, to evaluate the effect of the electroplated NiFe MFC on the noise of the sensor, the noise spectra of the sensor before and after the integration of the double-gap MFCs were both measured under 2.5 V bias, using an NI (National Instruments, Austin, TX, USA) PXIe-4492 data acquisition card with a sampling rate of 20,000. Additionally, the noise measurement was done when the sensor was saturated at the low resistance state and worked in the transition range. For the sensor without the double-gap MFC, the noise was measured under a magnetic field of 176.0 Oe and 22.0 Oe, and correspondingly an 8.0 Oe and −0.7 Oe magnetic field was applied in order to obtain the noise of the sensor with double-gap MFCs. In the saturation state, the sensor had minimal noise, and at the transition point the noise was maximal [27]. Therefore, we conducted a comprehensive evaluation of the effect of the double-gap MFC. The measured noise spectra are shown in Figure 7a,b. This figure presents that the noise spectra displayed the typical 1/*f* noise characteristics [28], and the noise voltage of the sensor changed a little after the introduction of double-gap MFC. Since the magnetic component is the main 1/*f* noise source for MTJ sensors and it is inversely proportional to the volume of magnetic material, the introduction of large MFC had little effect on the 1/*f* noise. The equivalent field noise (detectivity) for the device without and with the double-gap MFC at a DC magnetic bias field, which results in the highest sensitivity, is shown in Figure 7c. The result obviously shows that the detectivity of the sensor with the double-gap MFC was significantly improved. The sensitivity of our MTJ sensor with the double-gap MFC was 471 mV/V/Oe, and the equivalent field noise of the MTJ sensor in the paper with the double-gap MFC was 61.0 nT/√Hz at 10 Hz (in the transition range) and 1.7 nT/√Hz at 10 kHz. At present, the sensitivity is about 100 times larger than the anisotropic magnetoresistance (AMR) sensor, but the detectivity is not superior to the commercial AMR type sensor Honeywell HMC1021, whose detectivity is about 10 nT/√Hz at 10 Hz [27]. Fortunately, the low detectivity of our MTJ sensor was mainly caused by the high intrinsic noise of our MTJ device, which has demonstrated suppression to pT/√Hz by the design of a multilayer film, optimizing the micro-fabrication process and the annealing process [7]. What is more, the sensor can operate at a high frequency by the vertical motion modulation method, which would result in a drastic reduction of the 1/*f* noise [29,30].

## 5. Conclusions

In conclusion, a double-gap MFC was designed by setting a magnetic strip in the gap between the bridge arms. Theoretical and experimental results address that it is an effective strategy to further increase the overall performance of MTJ sensors. Encouragingly, the sensitivity of the half bridge MTJ sensor can be enhanced about 10 times by integrating the double-gap MFC. At the same time, the sensor noise insignificantly increased. The new sensors have a simple process compared with the structure of full bridge sensors. The method in this work provides a theoretical basis for MFC design, as well as guidance for the application of magnetic sensors.
